# Safety and efficacy of biological therapy with TNF-inhibitors and non TNF-inhibitors in a cohort of young adults affected by juvenile idiopathic arthritis (JIA): data from a single centre experience

**DOI:** 10.1186/1546-0096-9-S1-P273

**Published:** 2011-09-14

**Authors:** Ceri Lorenzo, Falcini Fernanda, Fiori Ginevra, Capannini Serena, Matucci Cerinic Marco

**Affiliations:** 1 Department of Internal Medicine, Rheumatology Section, Transition Clinic, University of Florence, Florence, Italy

## Aim

To evaluate safety and efficacy in young adults with JIA treated by TNF and non anti-TNF inhibitors.

## Methods

36 patients (F26-M10, median age 18,8 yrs) with JIA treated with anti-TNF (17 etanercept, 4 infliximab, 15 adalimumab) followed at Transition Clinic of Florence from January 2008 to December 2010 were enrolled in a observational, single-centre, retrospective study. 12 pts (4 etanercept, 4 infliximab, 4 adalimumab) failed to respond or did not tolerate the first therapy and switched to a second one. Moreover, 9 patients has received non anti-TNF drug (5 abatacept, 4 tocilizumab). In all, 53 treatments (19 etanercept, 5 infliximab, 20 adalimumab, 5 abatacept, 4 tocilizumab) were performed. Safety assessments were based on adverse events (AEs) report, divided in moderate AES (if infective events or injection site reactions have been occurred) and severe AES (including infusion reactions). Efficacy was assessed using the PedACR30/50/70 criteria.

## Results

Of the 36 patients treated with TNF-blockers, Ped ACR30/50/70 response was reached by 78%/67%/58% after 24 weeks, 72%/68%/60% after 48 weeks and 87%/87%/73% after 96 weeks of treatment. Of the 9 patients treated with abatacept or tocilizumab, PedACR30/50/70 response was reached by 89%/78%/67% after 24 weeks, 80%/80%/80% after 48 weeks and 75%/75%/75% after 96 weeks. 108 AEs (both moderate and serious) occurred in 26 patients (59%) treated with anti-TNF. Among non anti-TNF agents, 36 AEs occurred in 7 patients (78%), no one was serious and any patient leaved the biological treatment due to AEs.

## Conclusions

In our study, anti-TNF agents were well tolerated and provided clinically significant efficacy in young adults with JIA. In the refractory systemic form of JIA, tocilizumab seemed to be effective and safe.

